# Massive Gene Flux Drives Genome Diversity between Sympatric *Streptomyces* Conspecifics

**DOI:** 10.1128/mBio.01533-19

**Published:** 2019-09-03

**Authors:** Abdoul-Razak Tidjani, Jean-Noël Lorenzi, Maxime Toussaint, Erwin van Dijk, Delphine Naquin, Olivier Lespinet, Cyril Bontemps, Pierre Leblond

**Affiliations:** a Université de Lorraine, INRA, DynAMic, Nancy, France; b Institute for Integrative Biology of the Cell (I2BC), CEA, CNRS, University Paris-Sud, University Paris-Saclay, Gif-sur-Yvette, France; University of British Columbia

**Keywords:** *Streptomyces*, conjugation, gene transfer, plasticity, population

## Abstract

In this work, by comparing genomes of closely related individuals of *Streptomyces* isolated at a spatial microscale (millimeters or centimeters), we investigated the extent and impact of horizontal gene transfer in the diversification of a natural *Streptomyces* population. We show that despite these conspecific strains sharing a recent common ancestor, all harbored significantly different gene contents, implying massive and rapid gene flux. The accessory genome of the strains was distributed across insertion/deletion events (indels) ranging from one to several hundreds of genes. Indels were preferentially located in the arms of the linear chromosomes (ca. 12 Mb) and appeared to form recombination hot spots. Some of them harbored biosynthetic gene clusters (BGCs) whose products confer an inhibitory capacity and may constitute public goods that can favor the cohesiveness of the bacterial population. Moreover, a significant proportion of these variable genes were either plasmid borne or harbored signatures of actinomycete integrative and conjugative elements (AICEs). We propose that conjugation is the main driver for the indel flux and diversity in *Streptomyces* populations.

## Abstract (precis)

Horizontal gene transfer is a rapid and efficient way to diversify bacterial gene pools. Currently, little is known about this gene flux within natural soil populations. Using comparative genomics of *Streptomyces* strains belonging to the same species and isolated at microscale, we reveal frequent transfer of a significant fraction of the pangenome. We show that it occurs at a time scale enabling the population to diversify and to cope with its changing environment, notably, through the production of public goods.

## INTRODUCTION

*Streptomyces* organisms are prominent soil-dwelling bacteria found in all terrestrial ecosystems, typified by their complex differentiation cycle and their central roles in biogeochemical cycles and the homeostasis of the soil. They are known as the most prolific bacterial genus for the production of specialized metabolites (e.g., antibiotics and antifungals) and enzymes of high biotechnological and medical interest (1). In soil, these compounds are involved in interactions with the surrounding microbial communities and contribute to plant health and growth (2). Each species or strain was reported to produce one or only a few compounds, but access to the whole-genome sequences revealed a much more important biosynthetic gene reservoir (3, 4).

The genome of *Streptomyces* is remarkably large (6 to 12 Mb) and possesses one of the few linear bacterial chromosomes ever described. The genome is highly compartmentalized, with genes conserved across the species distributed at the center of the chromosome and variable genes in the terminal parts (5, 6). The biosynthetic gene clusters (BGCs) are consistently enriched in the variable part of the chromosome. The proportion of the flexible genome is impressive and is reflected at the genus level by a wide-open pangenome, i.e., the entire gene set of all species of the genus (7–9). The *Streptomyces* genomes can be completed by large linear and circular plasmids. Linear chromosomes and plasmids share the same invertron structure that consists of long terminal inverted repeats (TIRs) ending with telomeric sequences (10–12). In addition, the *Streptomyces* genomes are recombinogenic, as revealed either by the high frequency of spontaneous rearrangements of large DNA fragments (13) or by multilocus sequence analysis-based studies at the intra- and interspecific levels showing that recombination rates exceed those observed within many bacterial species (14).

Gene flux, i.e., the gain and loss of genetic material, crucially depends first on the access to exogenous information that can be achieved thanks to horizontal gene transfer (HGT) and second on the recombination capacities of the recipient genome that promotes the integration of genetic material in the genome (15). Regarding HGT, *Streptomyces* exhibits little talent for natural competence, and transducing phages have remained elusive (16, 17) despite extensive research. Thus, conjugation remains the only gene exchange mechanism described in *Streptomyces*, and the genomes of these bacteria are known to be rich in conjugative elements (integrated in the chromosome or of plasmid origin). Two types of conjugative elements coexist: integrated and conjugative element (ICE) depending on a type IV secretion system, and actinomycete ICE (AICE) whose transfer depends on a DNA translocase (TraB) (18, 19). The latter is the most widespread in *Streptomyces* and is capable of mobilizing chromosomal DNA in *trans*, i.e., not physically linked to the element (20).

These gene fluxes contribute to increase the flexibility of the genome and explain the diversity observed at the genus level. McDonald and Currie (21) recently showed that the *Streptomyces* genus is ancient (380 million years old) and that acquisition and retention of genes through HGT seem rare among nonclosely related lineages. Yet, nothing is known regarding the intensity and short-term impact of HGT at the population scale. Only some recent examples have shown that *Streptomyces* sharing an identical 16S rRNA gene sequences can exhibit differential phenotypes, including antimicrobial activities (22–24), suggesting rapid genome diversification.

Genome sequencing of closely related strains locally cooccurring has already revealed the existence of a large diversity in terms of gene content within different bacterial species (25–29). However, little is known regarding bacteria living in soil. Only a few examples are documented, and similarly, they seemed to indicate that variability in gene or allelic content can impact the ecology of soil bacteria. For instance, the polymorphism in quorum-sensing genes in Bacillus subtilis living in the same cubic centimeter impacted the ability to communicate and led to kin differentiation (30). In the same way, genomic comparison of two closely related groups of *Myxococcus* strains sampled at a centimeter scale enabled the identification of a 150-kb region involved in their sexual isolation (31). It seems thus relevant to assess genome diversification among soil inhabiting congenics, i.e., closely related strains, to unravel the genetic bases underlying the phenotypic diversity that supports niche differentiation and adaptation. The deeper the phylogenetic relationships (infraspecific level) are, the more recent the revealed events will be. Beyond analysis of 16S rRNA gene sequences, multilocus sequence analysis (MLSA), average nucleotide identity (ANI) across the genome, or phylogenomic tree reconstruction enable us to reach close phylogenetic relationships and to infer recent molecular events.

In soil ecosystems, the heterogeneity of the matrix constitutes a powerful driver of microbial growth and dynamics (32, 33). Biotic interactions in soil are most likely to occur between genetically related cells, produced by cell division, living close together on the same soil particle (34). Thus, bacteria in soil do not live as individual cells but are more likely structured in populations composed of closely related strains deriving from a recent common ancestor. The physical and phylogenetic proximities within these populations maximize the potentiality of gene exchanges. These latter reduce genetic isolation, disrupting the confinement of bacteria resulting from their reproduction scheme. However, the true extent of the gene flux that breaks clonality, and the impact of this on the function of natural bacterial populations, remains to be explored.

For that purpose, we undertook the first comparative genomics study of *Streptomyces* conspecifics. We favored the isolation of such closely related strains by sampling *Streptomyces* at the microscale and sequenced and compared their genomes. Next, we identified a high level of divergence in terms of the presence/absence of genes between them and mapped the events along the linear chromosome. This allowed us to identify recombination hot spots, some of which include BGCs. Next, we revealed the high prevalence of actinomycete integrative and conjugative elements (AICEs). We then consider the role of AICEs as motor of genome diversification and how this can later contribute to population functioning.

## RESULTS

### High levels of genetic diversity within a *Streptomyces* soil population.

**FIG 1:**
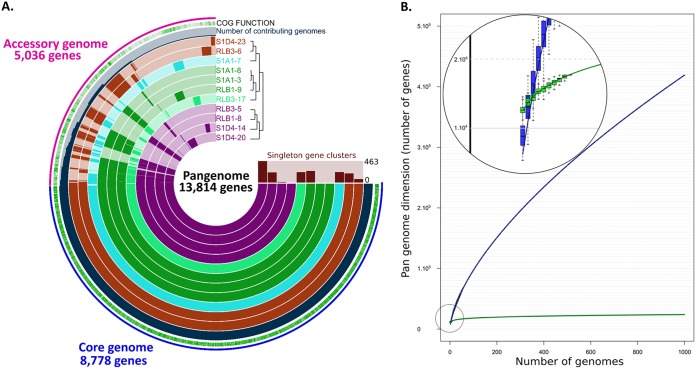
Pangenome analysis of the *Streptomyces* population. (A) Comparative Anvi’o genomic analysis of the 11 conspecific strains isolated in this study. The inner layers represent individual genomes organized regarding their phylogenetic relationships as indicated by the dendrogram. In the layers, dark colors indicate the presence of a gene group and light color its absence. The core (8,778 genes) and the accessory (5,036 genes) genomes are indicated in blue and pink, respectively, in the outmost layer. The blue layer represents the number of genomes among the population contributing to each gene group, and the green layer describes the gene groups in which at least one gene was functionally annotated using cluster of orthologous genes (COGs). (B) Comparison of the *Streptomyces* pangenome evolution at the genus and the population levels. The evolution of the pangenome of 59 complete *Streptomyces* genomes at the genus level is represented in blue. Its extrapolation to 1,000 genomes did not show any shift in the trend of the curve, indicating an open pangenome. The evolution of the population pangenome is represented in green. Its extrapolation rapidly reaches a plateau (see zoomed section).

To uncover microevolutionary processes, we isolated and sequenced the genomes of strains from a *Streptomyces* population inhabiting soil aggregates. For that purpose, four grains of soil, on the order of cubic millimeters in size and distant from each other from 2 cm to a maximum of 8 cm, were sampled from a clod of soil from which we isolated 129 strains. Based on identical 16S rDNA-coding gene sequences and a high degree of identity (>99.8%, nucleotide [nt]) measured with a five-gene multilocus sequence typing (MLST) scheme (see Fig. S1A in the supplemental material), we identified 32 highly related strains at the infraspecific level. The closest species to our group of strains was Streptomyces olivochromogenes, with in average 99.93% identity in the 16S rRNA gene and 94.5% ANIblast with the strain DSM40451. The genomes of 11 strains, representative of the different MLSTs identified in the cluster 6 of the population (Fig. S1A), were fully sequenced by a combination of Nanopore (Oxford Nanopore Technologies) and Illumina technologies (see Table S1). For each strain, a single scaffold for each replicon (i.e., chromosome and plasmids) was obtained with high-quality standards enabling full genome comparison. Phylogenomic tree construction (Fig. S1B) and calculation of ANIb percentages (ranging from 98.68% to 99.99%) between all isolates confirmed that they belonged to the same species and diverged recently from a common ancestor. The genomes ranged from 11.75 Mb to 12.44 Mb in size, positioning our strains among the biggest bacterial sequenced genomes (35). The chromosomes are linear with terminal inverted repeats (TIRs) of various sizes (from 311 kb to 587 kb) (see Table S2), as previously reported at the intra- and interspecific levels (12, 36). Following genome annotation (RAST), we determined that the pangenome of our population consisted of 13,814 genes (Fig. 1A). By extrapolating, we found that it reaches a maximum of 23,772 genes by 1,000 genomes. In contrast, for 11 strains randomly chosen in the genus, the pangenome was found to be approximately twice that of the population (23,672 versus 13,814) (Fig. 1B). Regarding the population pangenome, approximately one-third (5,036 genes) was not shared across the population and constituted the accessory genome (Fig. 1A). No two strains shared the same gene content; the closest differed by 12 predicted genes, while the two more distant isolates possessed 1,014 and 1,393 specific genes. Approximately one-quarter (27.6%; 1,392 genes) of the accessory genes were harbored on circular or linear extrachromosomal elements (98.8 kb to 394 kb), which were predicted *in silico* or experimentally observed by pulsed-field gel electrophoresis (PFGE) (see Fig. S2 and Table S2). These elements were found in single strains, and their mobile nature highlights the role of HGT as a driver of specificity, enabling the transfer of numerous genes simultaneously.

10.1128/mBio.01533-19.1 FIG S1 Phylogenetic trees. (A) Multilocus sequence typing (MLST) tree of isolated strains. The tree is constructed by neighbor joining with a K2P distance correction and 100 bootstrap replicates. It is based on the concatenated sequence alignment of five loci (*atpD*, *gyrB*, *recA*, *rpoB*, and *trpB*). The final dataset has 3,178 positions in total. The scale bar indicates 1% estimated sequence divergence. Clades are represented by colored boxes. Strains whose genomes were sequenced are represented in red inside clade 6. (B) Phylogenomic analysis was performed with 5,213 genes (5,149,602 nucleotide positions) of the core genome of the population shared with Streptomyces avermitilis, which was used as a root. The tree was built in maximum likelihood with a GTR model and 100 bootstrap replicates. Download FIG S1, PDF file, 0.6 MB. Copyright © 2019 Tidjani et al. 2019 Tidjani et al. This content is distributed under the terms of the Creative Commons Attribution 4.0 International license.

10.1128/mBio.01533-19.2 FIG S2 Extrachromosomal DNA content. PFGE analysis of undigested DNA of strains RLB1-9, S1D4-14, and S1D4-20 after growth at 30°C for 36 h. Running conditions were 6 V·cm^−1^ for 20 h with ramped pulse times from 40 s to 160 s. Download FIG S2, PDF file, 0.4 MB. Copyright © 2019 Tidjani et al. 2019 Tidjani et al. This content is distributed under the terms of the Creative Commons Attribution 4.0 International license.

10.1128/mBio.01533-19.5 TABLE S1 Summary table of genome sequencing data. For Illumina sequencing, all strains were sequenced using 250-nt paired ends except for RLB1-9 (*) and RLB1-8 (**), which were sequenced using 75-nt and 300-nt paired ends, respectively. Download Table S1, PDF file, 0.4 MB. Copyright © 2019 Tidjani et al. 2019 Tidjani et al. This content is distributed under the terms of the Creative Commons Attribution 4.0 International license.

10.1128/mBio.01533-19.6 TABLE S2 Table of genomic data. Download Table S2, PDF file, 0.5 MB. Copyright © 2019 Tidjani et al. 2019 Tidjani et al. This content is distributed under the terms of the Creative Commons Attribution 4.0 International license.

### Genome diversity is unevenly distributed in numerous indels along the linear chromosome.

Pairwise genome comparisons (*n* = 65) among the 11 sequenced genomes enabled us to identify disruptions in chromosomal synteny with insertion or deletion events (indels) of a minimum of one gene. The numbers of breaks differing between two strains ranged from 1 to 124 and increased linearly with the phylogenetic distance, indicating that they accumulate over time (see Fig. S3). When a break was shared by at least two strains, we considered it a single genetic event that may have been vertically inherited. This allowed consideration of 452 independent events that occurred during the population diversification (see Table S3). In extreme cases, the closest strains differed by only two events, while the most distinct strains differed by 235.

10.1128/mBio.01533-19.3 FIG S3 Correlation between phylogenetic distance and synteny breaks. The phylogenetic distance was determined on the core genome and is expressed as a percentage of dissimilarity. Each dot represents the number of synteny breaks encountered in a pairwise comparison. The dotted line represents the correlation curve (*R*^2^ = 93.09). Download FIG S3, PDF file, 0.4 MB. Copyright © 2019 Tidjani et al. 2019 Tidjani et al. This content is distributed under the terms of the Creative Commons Attribution 4.0 International license.

10.1128/mBio.01533-19.7 TABLE S3 Distributions of indels events along the chromosome. The chromosome of strain RLB1-8 was chosen as a reference for pairwise comparisons. The position of an indel is indicated according to the position of its first coding DNA sequence (CDS) in RLB1-8. This position corresponds to the CDS number in RLB1-8. Cells with identical colors indicate that the strains share the same indel at this position. The numbers in the cells indicate the number of CDSs in the indel. *, presence of an AICE in the indel; #, presence of an ICE in the indel. Download Table S3, PDF file, 0.8 MB. Copyright © 2019 Tidjani et al. 2019 Tidjani et al. This content is distributed under the terms of the Creative Commons Attribution 4.0 International license.

**FIG 2:**
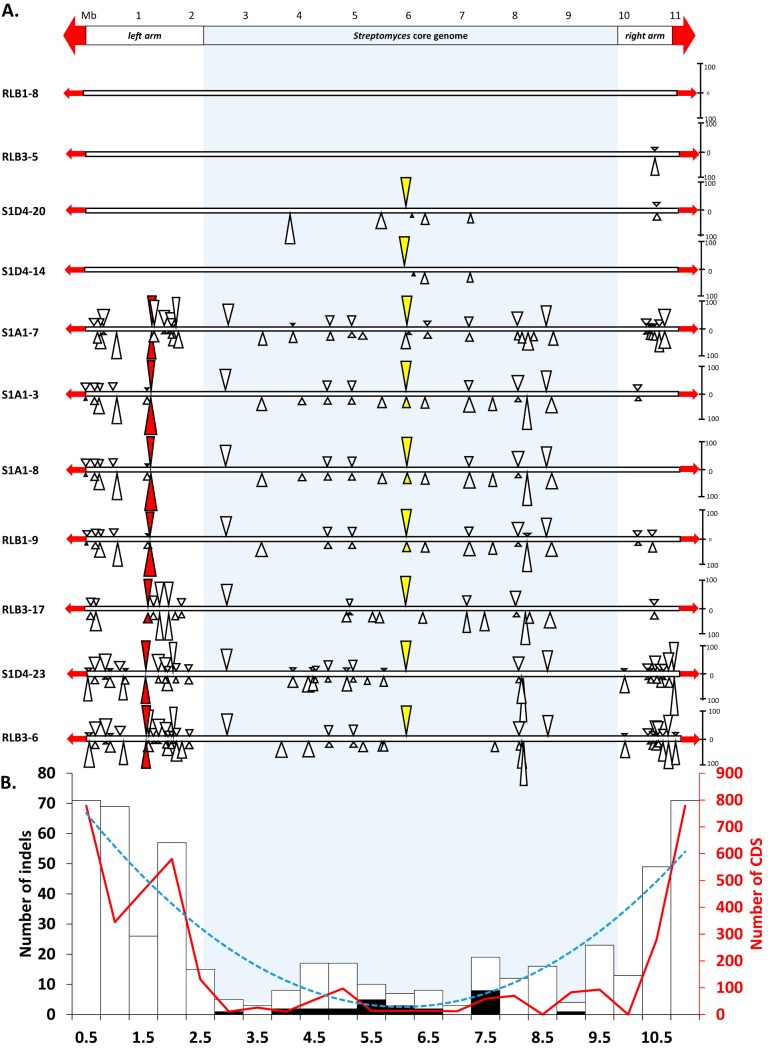
Distribution of insertion and deletion events along the linear chromosome of *Streptomyces*. (A) The scheme at the top represents the *Streptomyces* chromosome with a megabase scale. The position of the core genome of the *Streptomyces* genus is highlighted by a light gray frame. The terminal inverted repeats (TIRs) are shown as red arrows. The bottom portion shows illustrations of pairwise genome comparisons (among the 10 possible pairs) within the population using strain RLB1-8 as a reference. Rectangles represent the linear chromosome of each strain. The strains are ordered from top to bottom relative to their phylogenetic distance to the reference. Triangles above the chromosome represent insertions in the reference strain, while triangles below correspond to insertions in the compared strain. For the sake of clarity, only the insertions of at least 10 predicted genes are shown. The height of a triangle reflects the number of genes involved in the insertion (the scale on the right of the chromosome indicates the number of genes). The colored triangles correspond to examples of insertion hot spots (the yellow and red triangles correspond to the hot spots depicted in Fig. 3 and 4, respectively). (B) The scheme represents the distribution of all the indel events identified within the population by genome pairwise comparisons. Each histogram bar corresponds to the number of indels within a 0.5-Mb window. The proportion of ICE/AICE insertions within a window is shown in black. The dotted blue line (“smiley” curve) corresponds to the polynomial trend curve (order 2, *R*^2^ value) of the indel distribution. The red curve shows the number of genes involved in indels within a window.

**FIG 3:**
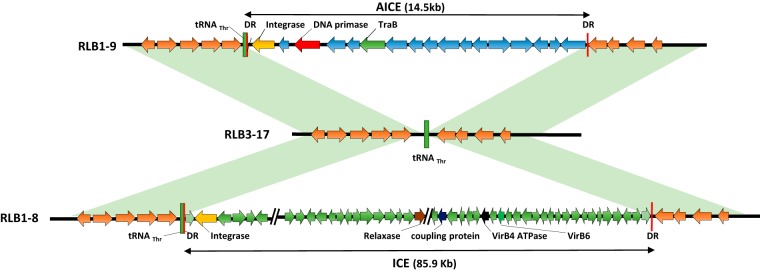
ICE/AICE insertion hot spot. The scheme is illustrating a hot spot for conjugative and integrative element insertion. Two different elements, AICE and ICE, are inserted in the tRNA_Thr_ genes of strains RLB1-9 and RLB1-8, respectively. The tRNA_Thr_ insertion site remains empty in RLB3-17. Direct repeats (DR) flanking the mobile element are represented in red (43 nucleotides for RLB1-9 and 42 nucleotides for RLB1-8). Only the key genes used to identify the elements are shown. Regions in light green correspond to syntenic regions.

**FIG 4:**
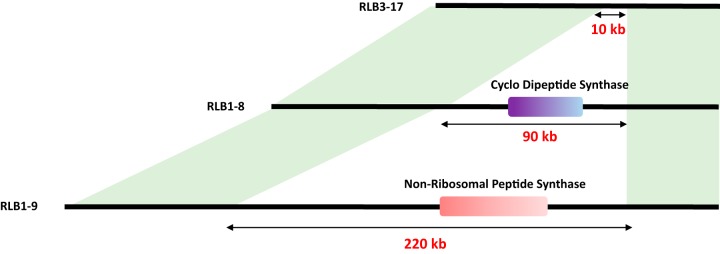
Identification of biosynthetic gene clusters in a variability hot spot. The scheme is illustrating a hot spot of variability observed between three strains (RLB3-17, RLB1-8, and RLB1-9). In two of them, different biosynthetic gene clusters were predicted by antiSMASH: one including a tRNA-dependent cyclodipeptide synthase in RLB1-8 and one including a nonribosomal peptide synthase in RLB1-9. Regions in light green correspond to syntenic regions.

Comparative genomics of the 11 strains shows that indels were scattered along the chromosome but tended to form hot spots (Fig. 2A) with locations where at least three distinct gene assemblies were encountered (Fig. 3 and 4). Remarkably, indel distribution was heterogeneous along the chromosome, with increased density in the terminal regions of the chromosome, and was exaggerated as the phylogenetic distance of strains increased (e.g., RLB1-8 versus RLB3-6). Mapping the core genome of the *Streptomyces* genus (971 genes [J.N. Lorenzi, A. Thibessard, O. Lespinet, P. Leblond, unpublished]) identified the two distal positions, which delineate the core region from chromosomal arms of approximately 2.4 Mb and 1.4 Mb which are devoid of conserved genes on the left and right arms, respectively. The indel density was approximately 5-fold higher in the arms than in the core region (Fig. 2B). Moreover, the compilation of all the pairwise comparisons shows that the density of indels increases from these positions toward the ends of the chromosome, forming a gradient of events. Although the size of the indel in the core region appears larger than in the arms, the total numbers of variable genes follow the same trend, i.e., a strong increase in the arms. The highest variability was noticed at the very ends of the chromosome, as no two strains were identical in terms of the size of their terminal inverted repeats (Table S2). As previously reported, this variability might result from the formation of indels in the TIRs followed by chromosome arm replacement (36–38).

### Conjugative elements as the motor of rapid genome diversification.

Genome mining for the presence of integrative and conjugative elements in our population allowed the identification of one ICE and 25 AICEs. These 26 elements are likely to be functional (i.e., excisable and self-transmissible), since all the key functions (integrase and Tra translocase) and *cis* sequences (flanking *att* sites) are present in each of them. Each genome has on average five elements, which make our population particularly rich compared to two elements found on average in a previous study on *Actinobacteria* (18). All the identified elements in our population were present in indels, meaning that they were not part of the core genome, highlighting their remarkable dynamics among the population. They were mainly inserted within the central region of the chromosome (Fig. 2B) and tended to form hots pots with several distinct elements being aggregated in the same target sequence. As illustrated in Fig. 3, the same tRNA target site was occupied by two distinct elements; while an AICE was present in RLB1-9, an ICE was observed in RLB1-8. In the third strain (RLB3-17), the target site was empty, which suggests that at least two of these strains experienced different conjugative events. While conjugative transfer is acknowledged as a powerful driver of diversity in telluric bacteria, their mobility in the soil remains difficult to demonstrate (39, 40). Here, the fact that each strain of the population is unique in terms of AICE/ICE content suggests that the exchanges are intense in the soil.

### HGT fosters social cohesiveness of bacterial population.

*Streptomyces* organisms are prolific antibiotic producers, and specialized metabolite (SM) production has already been reported to be highly variable among related strains of *Streptomyces* (22–24). We tested our *Streptomyces* isolates in pairwise inhibitory and resistance bioassays and found that none was able to inhibit its conspecifics. On the other hand, some but not all produced antimicrobial activity against a *Bacillus* strain isolated from the same soil, named “killer strains.” To correlate the inhibitory phenotype with the presence/absence of biosynthetic gene clusters (BGCs), we performed comparative genomic analyses using antiSMASH (41). We identified 51 unique BGCs within the population, each strain possessing around 35 clusters (not shown). Interestingly, some of these BGCs were present in a variability hot spot (Fig. 4), with three strains exhibiting different gene content at these locations. The strain RLB3-17 contained no BGC, while RLB1-8 harbored a cyclodipeptide synthase BGC and RLB1-9 a nonribosomal peptide synthetase (NRPS) BGC. The disruption of a key gene belonging to the NRPS BGC (Fig. 4) only present in the *Streptomyces* killer strains (including RLB1-9) abolished the inhibitory activity against *Bacillus* (see Fig. S4), proving that this BGC is responsible for the antimicrobial activity of the killer strains under these conditions.

10.1128/mBio.01533-19.4 FIG S4 Abolition of inhibitory activity of the strain RLB1-9 by NRPS disruption. Spore suspensions of RLB1-9 (A) and of the RLB1-9 NRPS mutant (B) were incubated 5 days to enable the diffusion of specialized metabolites. They were then overlaid with a *Bacillus* indicator strain. In panel A, the growth of *Bacillus* is inhibited by RLB1-9 as scored by the clearance zone, while the NRPS mutant in panel B showed no inhibitory activity. Download FIG S4, PDF file, 0.4 MB. Copyright © 2019 Tidjani et al. 2019 Tidjani et al. This content is distributed under the terms of the Creative Commons Attribution 4.0 International license.

## DISCUSSION

In this work, we isolated *Streptomyces* conspecifics at a soil microscale, and we revealed by genome comparison the extent of the massive gene flux that occurs in this natural population. This is reflected by a large pangenome, with almost clonal strains harboring strain-specific gene content. The size of the pangenome is influenced by three parameters: (i) the capacity to capture new DNA sequences, (ii) ecological selection of adaptive gene sets, and (iii) the evolutionary time enabling the accumulation of genome divergence (42). The fact that the population pangenome appears reduced in comparison with that of the genus (Fig. 1B) might reflect a constrained flux of genetic information within the microhabitat that is dependent of the diversity of the incoming information mostly arising from sister strains. However, the genetic diversity remained high in our population, implying an intense rate of gain and loss of genome regions over a short evolutionary time.

In *Streptomyces*, we have shown recently that repair of chromosomal double-strand breaks (DSB) experimentally induced in the terminal regions triggers the formation of chromosomal rearrangements (43). The formation of short insertions was also associated with DSB repair depending of nonhomologous end joining (NHEJ). Although the underlying mechanisms are mostly unknown, the plasticity of the subtelomeric regions appears as a hallmark of chromosome linearity in many organisms, from the limited range of bacterial taxa harboring linear chromosomes (44) to eukaryotes (45), including humans (46). In *Streptomyces*, tolerance to DNA rearrangements in the terminal regions could find its origins in the ancestral chromosomal linearization event. It is assumed that the ancestral actinomycete chromosome (i.e., harboring the ancestral core genes) was circular and linearized by recombination with an incoming linear replicon, leading to the addition of chromosomal arms harboring mostly contingency genes (47). However, if the ancestral gain of chromosomal arms can explain the tolerance to DNA rearrangements, it does not shed light on the evolutionary mechanisms that led to the contemporary variability of the *Streptomyces* genome (8, 9, 48). With respect to this, previous work has shown that chromosomal regions separating the core region from chromosomal arms were riddled with short indels (1 to 10 genes) forming a gradient toward the terminal ends (5). We speculate that the indels, observed at the population level, when cumulated over evolutionary times, gave rise to the genetic compartmentalization observed within the genus (5).

Considering the high prevalence and plasticity of AICEs, we further propose that conjugative mechanisms are responsible for the massive gene flux observed in indels. To mobilize DNA, the translocase TraB binds to specific 8-mer repeats (named *clt* for *cis* acting locus of transfer) present on the AICE and assumed to be also present on the chromosome (*clt* like). Thus, beyond promoting self-transfer of the elements, TraB is able to transfer chromosomal markers in *trans* (20). This property was characterized and exploited in early studies on *Streptomyces* genetics (49). While conjugative transfer of chromosomal DNA was proved to be efficient under laboratory conditions (49, 50), the extent (frequency and size of transferred DNA stretches) remains to be elucidated. The indels ranging from 1 to 241 genes give an indication of the size of the chromosomal DNA stretches that may be transferred through conjugational processes. This is reminiscent of the distributive conjugal transfer in mycobacteria that enables the transfer of chromosomal fragments creating chimeric chromosomes in the recipient cells (51, 52). It is tempting to speculate that several DNA fragments could be transferred at once and generate multiple and diverse recombination events in the recipient *Streptomyces* hyphae.

At the ecological level, genome plasticity increases the functional potential at the strain but also the population level. For instance, it can enable the specific expression of specialized metabolites that can constitute public goods. Bacterial populations (i.e., sympatric closely related strains) can act as social units, meaning that their competitive interactions are more intense toward individuals not belonging to the population rather than between members of the population. Variability of SM production and resistance has already been reported as a driver of ecological cohesive units in *Vibrio* (53) and *Streptomyces* (23). Accordingly, differential SM production and resistance can be used as a proxy to understand the ecological dynamics of the bacterial population (53–55). Here, we confirm that the intense gene flux that *Streptomyces* conspecifics experienced promotes this variability, as previously proposed by Vetsigian et al. (23).

The production of SM within a bacterial population may underpin two principal ecological outcomes, bearing in mind that actual interactions in the soil may be more complex or different than under laboratory conditions. The production of antimicrobial activity and its respective resistance may benefit the sole carrier of the antibiotic production gene and mediate competition with conspecifics as well as with other species. Alternatively, an antibiotic produced by only some individuals of the population where the rest are resistant supports the hypothesis that antibiotics can constitute public goods, benefiting nonproducing but resistant conspecifics. Since none of our isolates was able to inhibit its conspecifics, it supports the hypothesis that they formed a cooperative population. The acquisition of the capacity to produce antimicrobial activity together with the capacity to resist its toxicity can result from a single HGT event thanks to the clustering of biosynthetic and resistance genes in the same genetic region, which is a typical trait of BGCs in actinomycetes. Therefore, the emergence of individuals able to overcome their conspecifics by producing a lethal activity is easily achieved by HGT. However, as we show in this work, our *Streptomyces* population experienced a massive and intense gene flux, which, in addition to favoring the acquisition of BGCs by individuals can also promote a rapid dissemination of all or parts of BGCs to the rest of the population. The replacement of a complete BGC obviously diversifies the antimicrobial arsenal, while its loss accompanied or not by that of the resistant determinant can result in a high intrapopulational SM variability. Further, recombination within or between BGCs may be enhanced thanks to their highly redundant structure (i.e., modular organization of NRPS and polyketide synthase [PKS]) and foster the emergence of new BGCs and the production of new antimicrobial compounds. The intense turnover of the BGC and resistance gene sets would constantly modulate the interactions within the population and, through this dynamic state, favor the social cohesiveness of the population.

## MATERIALS AND METHODS

### Strain isolation and maintenance.

Four grains of soil on the order of cubic millimeters in size and distant from each other from 2 cm to a maximum of 8 cm were sampled from a clod of soil collected in the Montiers-sur-Saulx forest in France (GPS coordinates: 48°32′37.248′′N, 5°18′21.946′′E) and stored for 48 h at 4°C before processing. For bacterial isolation, each soil aggregate was dissolved 1:100 (wt/vol) in sterile water by vortexing for 15 min. Serial dilutions from 10^−1^ to 10^−3^ were spread on *Streptomyces* isolation medium (SIM) (56), and bacterial colonies showing a typical *Streptomyces* phenotype were randomly picked after 7 days of incubation at 30°C. After three consecutive subcultures on mannitol soy agar plates (57), strains were stored as spore suspensions in 20% glycerol (57).

### Genome sequencing, annotation, and DNA manipulation.

Total genomic DNA was isolated as previously described by Kieser et al. (57). Each genome was sequenced by one-dimension MinION (Oxford Nanopore Technologies, UK) (mean coverage of 108×) and further corrected with MiSeq sequencing (Illumina, CA, USA) (mean coverage 50×), enabling the acquisition of each chromosome in one scaffold and the identification of extrachromosomal elements when present. Sequencing and assembling were performed via the I2BC NGS platform (France). Genome sequencing data and genome accession numbers are listed in Table S2 in the supplemental material. Coding sequence prediction and annotation were performed using the NCBI Prokaryotic Genome Annotation Pipeline (58). Genes involved in SM biosynthesis were predicted with antiSMASH (41).To disrupt a gene cluster putatively involved in the biosynthesis of a NRPS in strain RLB1-9, a 897-bp sequence internal to the peptide synthetase gene (open reading frame [ORF]-07845) was amplified and cloned into the suicide vector pIJ8668 to give pIJ8668-*intORF-07845* (59). Intergeneric Escherichia coli/*Streptomyces* conjugation (60) followed by selection for apramycin resistance (pIJ8668) allowed us to select NRPS-encoding gene disruption by homologous recombination. Independent mutant clones (i.e., from distinct transformation assays) were tested for their antimicrobial activities. Pulsed-field gel electrophoresis (PFGE) was performed on whole genomic DNA in agarose plugs as described previously (61).

### Phylogenetic analyses.

16S rRNA genes and MLST genes (*atpD*, *gyrB*, *recA*, *rpoB*, and *trpB*) were amplified using primers and PCR conditions described by Rintala et al. (62) and Guo et al. (63), respectively. Neighbor-joining phylogenetic trees were built using single or concatenated MLST gene (3,178 positions) sequences with a Kimura’s two-parameter distance correction and 100 bootstrap replicates (MEGA7 software [64]). Phylogenomic analyses were performed with the nucleotidic sequences of the core genome identified by a Best BLAST Hit analysis (65), subsequently aligned with Muscle (v3.8.31 [66]), and corrected with Gblocks (67). From the 8,778 genes of the population core genome, we selected the 5,213 genes (5,149,602 nucleotide positions) shared with Streptomyces avermitilis, the reference strain used as a root in the phylogenetic reconstruction. Maximum likelihood phylogenomic tree (GTR, 100 bootstrap replicates) was built with RaxML (68). Python scripts (unpublished) were used to calculate the average nucleotide identity (ANIb) (69) and to identify and to extrapolate pangenomes. Pangenome visualization was performed using Anvi’o (70), and extrapolation graphs were computed with the UpsetR package in script R. Functional AICEs and ICEs were identified first by searching signature genes (replication, transfer, excision, and integration) using BLASTP (65) as described by Bordeleau et al. (18) and then by identifying direct repeats flanking the element. Breaks of synteny were identified using an in-lab developed software (unpublished and available upon request).

### Antibacterial activity assays.

Drops of *Streptomyces* spore suspensions (5 μl of spore stock, 10^7^ spores per ml) were spotted and incubated for 5 days at 30°C on GA medium plates (54). Plates were overlaid with a Luria-Bertani top (0.4% agar), inoculated with *Bacillus* (optical density at 600 nm [OD_600_] of 0.1), and incubated at 4°C for 2 h and then at 30°C overnight. Growth inhibition of *Bacillus* was assessed by the observation of a zone of clearance. To test *Streptomyces*-*Streptomyces* inhibitory capacities, spores of a first strain were spotted on mannitol soy medium plates for a 3-day incubation at 30°C. Then, a similar deposit of spores of the second strain (indicator) was adjacency spotted (5 mm) and incubated for 5 days at 30°C. The absence of phenotypic variation in comparison with control strains inoculated alone was considered a neutral interaction, and growth or sporulation alterations were recorded as antagonism.

### Accession number(s).

The genome accession numbers for the strains in this study are CP041650 to CP041654 and CP041601 to CP041613.
