# Supplementary figures and images for: Massive Gene Flux Drives Genome Diversity between Sympatric Streptomyces Conspecifics
Source: mBio. 2019 Sep 3;10(5):e01533-19. doi: 10.1128/mBio.01533-19 (PMC6722414; doi:10.1128/mBio.01533-19)

Fig. S1.

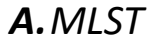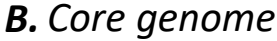

Supplement: FIG S1 [file mBio.01533-19-sf001.pdf]

Fig. S2

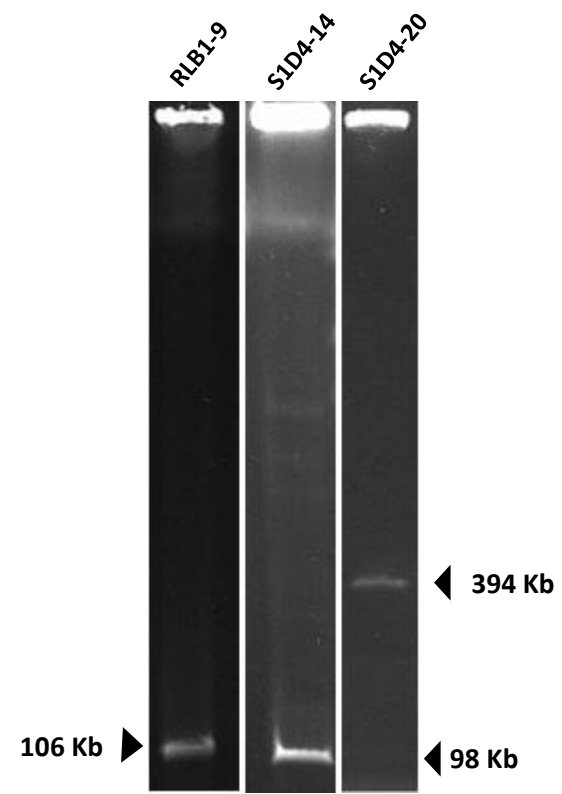

Supplement: FIG S2 [file mBio.01533-19-sf002.pdf]

Fig. S3

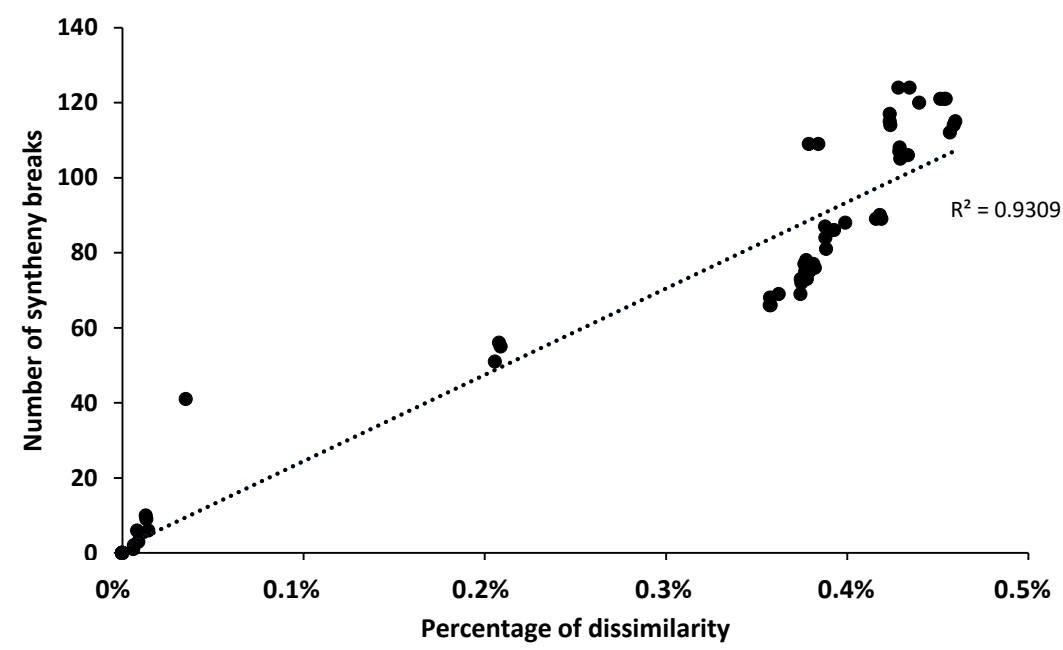

Supplement: FIG S3 [file mBio.01533-19-sf003.pdf]

Fig. S4

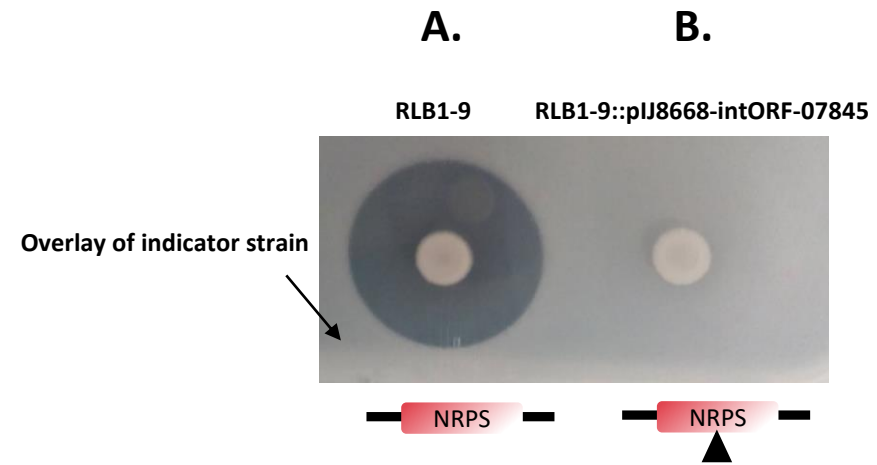

Supplement: FIG S4 [file mBio.01533-19-sf004.pdf]
