# Supplementary material for: Massive Gene Flux Drives Genome Diversity between Sympatric Streptomyces Conspecifics
Source: mBio. 2019 Sep 3;10(5):e01533-19. doi: 10.1128/mBio.01533-19 (PMC6722414; doi:10.1128/mBio.01533-19)
Supplement: TABLE S1 [file mBio.01533-19-st001.pdf]

Table S1

	Illumina sequencing		Nanopore sequencing				
Strain	Number of reads	Coverage	Number of reads	Number of bases	Coverage	Number of contigs	Estimated genome size
RLB1-8	4,492,734**	150	655,492	1,794,236,654	150	2	11,652,134
RLB1-9	9,166,845*	107	88,694	488,157,099	41	3	12,146,841
RLB3-5	1,598,054	53	144,521	673,009,805	56	10	11,697,055
RLB3-6	1,637,136	55	299,155	618,705,318	52	5	12,454,816
RLB3-17	1,988,311	66	202,455	603,258,039	50	1	12,023,175
S1A1-3	1,621,592	54	197,777	612,096,268	51	7	11,988,482
S1A1-7	1,752,105	58	533,299	876,735,911	73	18	11,820,843
S1A1-8	1,595,659	53	780,962	632,637,759	53	16	12,040,123
S1D4-14	1,397,227	47	2,065,804	4,195,872,938	350	5	11,934,153
S1D4-20	1,663,586	55	1,425,070	3,098,746,284	258	5	12,255,226
S1D4-23	1,771,880	59	400,348	735,306,850	61	8	12,060,819
