# Supplementary material for: Massive Gene Flux Drives Genome Diversity between Sympatric Streptomyces Conspecifics
Source: mBio. 2019 Sep 3;10(5):e01533-19. doi: 10.1128/mBio.01533-19 (PMC6722414; doi:10.1128/mBio.01533-19)
Supplement: TABLE S2 [file mBio.01533-19-st002.pdf]

Table S2

Strain	Replicon size	Genome size	Number of Coding Sequence	G+C %	Terminal Inverted Repeat size (kb)	Accession number
RLB1-8	11,765,340	11,765,340	10,538	70.2	357	CP041650
RLB1-9	11,940,408	12,200,709	10,729	70.2	311	CP041654
pRLB1-9.1	154,158[C]		181	69,0	-	CP041653
pRLB1-9.2	106,143[L]		120	68.7	24	CP041652
RLB3-5	11,898,970	11,898,970	10,632	70.2	365	CP041651
RLB3-6	12,338,263	12,448,281	11,088	70.1	587	CP041602
pRLB3-6.1	110,314[C]		100	70.6	-	CP041601
RLB3-17	12,023,175	12,023,175	10,809	70.2	451	CP041610
S1A1-3	12,042,091	12,042,091	10,801	70.2	393	CP041611
S1A1-7	11,713,151	12,005,504	10,44	70.3	513	CP041604
pS1A1-7.1	292,353[C]		251	69.7	-	CP041603
S1A1-8	12,036,971	12,036,971	10,806	70.2	394	CP041612
S1D4-14	11,723,487	11,934,498	10,472	70.2	369	CP041607
pS1D4-14.1	112,196[L]		117	68.7	0	CP041605
pS1D4-14.2	98,815[C]		150	69.1	-	CP041606
S1D4-20	11,851,257	12,245,276	10,642	70.2	373	CP041609
pS1D4-20.1	394,019[L]		372	69.1	68	CP041608
S1D4-23	12,057,712	12,057,712	11	70.2	421	CP041613

[L] indicates an extrachromosomal replicon detected by PFGE.

[C] indicates a circular extrachromosomal replicon identified in *silico* and consistently not visible in PFGE.
