# Supplementary material for: Massive Gene Flux Drives Genome Diversity between Sympatric Streptomyces Conspecifics
Source: mBio. 2019 Sep 3;10(5):e01533-19. doi: 10.1128/mBio.01533-19 (PMC6722414; doi:10.1128/mBio.01533-19)
Supplement: TABLE S3 [file mBio.01533-19-st003.pdf]

Table S3

CDS position in RLB1-8 (Reference)		Strains									
	RLB1-8	S1D4-23	RLB1-9	S1A1-3	S1A1-8	S1A1-7	RLB3-6	RLB3-17	RLB3-5	S1D4-14	S1D4-20
61	3	3	3	3	3	3	49	3	3	3	3
82	0	0	3	3	3	0	0	0	0	0	0
97	4	4	4	4	4	4	2	4	4	4	4
110	3	3	3	3	3	3	3	3	3	3	3
135	64	64	64	64	64	64	86	64	64	64	64
136	4	4	4	2	2	4	4	2	4	4	4
147	52	52	52	52	52	12	52	52	52	52	52
183	32	19	32	32	32	32	32	32	32	32	32
190	3	0	0	0	0	3	3	0	3	3	3
196	3	3	3	8	3	3	3	3	3	3	3
199	0	0	6	0	6	0	0	0	0	0	0
206	9	9	9	9	9	41	41	9	9	9	9
220	2	2	2	2	2		5	2	2	2	2
231	8	8	8	8	8	4	8	8	8	8	8
235	9	9	12	12	12	9	9	12	9	9	9
247	0	0	0	0	0	9	0	0	0	0	0
249	3	3	0	0	0	3	3	3	3	3	3
259	3	5	3	3	3	5	5	3	3	3	3
265	11	1	11	11	11	4	4	11	11	11	11
282	11	4	5	5	5	4	4	11	11	11	11
321	1	7	1	1	1	1	7	1	1	1	1
339	0	0	0	0	0	0	0	0	0	6	0
341	35	64	35	35	35	35	35	5	35	35	35
343	4	4	4	4	4	2	4	4	4	4	4
355	13	13	1	1	1	13	13	13	13	13	13
365	5	5	5	5	5	5	5	5	5	5	5

Table S3 - continued

CDS position in RLB1-8 (Reference)	Strains										
	RLB1-8	S1D4-23	RLB1-9	S1A1-3	S1A1-8	S1A1-7	RLB3-6	RLB3-17	RLB3-5	S1D4-14	S1D4-20
376	4	4	4	4	4	4	4	5	4	4	4
378	2	2	3	3	3	2	2	2	2	2	2
384	1	1	3	3	3	1	1	1	1	1	1
422	1	57	1	1	1	1	57	1	1	1	1
448	0	3	0	0	0	0	0	0	0	0	0
455	5	5	5	5	5	5	5	6	5	5	5
465	0	0	4	4	4	0	0	0	0	0	0
467	3	3	6	6	6	3	3	1	3	3	3
469	2	2	2	2	2	3	2	2	2	2	2
474	5	1	5	5	5	5	1	5	5	5	5
476	0	0	2	2	2	3/0	0	3/0	0	0	0
510	6	6	2	2	2	6	6	2	6	6	6
513	3	0	3	3	3	3	0	3	3	3	3
522	5	2	5	5	5	5	2	5	5	5	5
525	2	2	2	2	2	4	2	2	2	2	2
530	0	0	5	5	5	0	0	0	0	0	0
531	3	4	3	3	3	3	4	3	3	3	3
537	20	10	20	20	20	20	10	20	20	20	20
543	14	14	12	12	12	5	14	12	14	14	14
565	0	0	0	0	0	16	0	4	0	0	0
578	2	2	2	2	2	2	5	2	2	2	2
582	0	8	0	0	0	0	4	0	0	0	0
584	3	3	3	3	3	3	4	3	3	3	3
600	2	2	3	3	3	2	2	3	2	2	2
610	14	14	37	37	37	33	14	33	14	14	14
616	1	1	1	1	1	1	3	1	1	1	1
626	3	3	2	2	2	2	3	3	3	3	3

Table S3 - continued

CDS position in RLB1-8 (Reference)		Strains									
	RLB1-8	S1D4-23	RLB1-9	S1A1-3	S1A1-8	S1A1-7	RLB3-6	RLB3-17	RLB3-5	S1D4-14	S1D4-20
630	3	3	3	3	3	3	3	3	3	3	3
639	0	0	3	3	3	0	0	0	0	0	0
643	35	2	35	35	35	2	2	35	35	35	35
674	5	5	5	5	5	5	5	3	5	5	5
693	3	3	3	3	3	3	4	3	3	3	3
701	1	1	1	4	4	1	1	1	1	1	1
705	4	4	4	2	2	4	4	2	4	4	4
712	2	4	2	2	2	2	4	2	2	2	2
731	1	17	1	1	1	1	17	1	1	1	1
755	2	7	3	3	3	2	3	2	2	2	2
760	3	1	1	1	1	3	1	3	3	3	3
766	7	7	4	4	4	4	7	4	7	7	7
769	4	7	4	4	4	4	7	4	4	4	4
779	0	3	0	0	0	0	3	0	0	0	0
810	6	1	6	6	6	6	1	6	6	6	6
852	1	6	1	1	1	1	6	1	1	1	1
855	4	5	4	4	4	4	5	4	4	4	4
930	6	4	4	4	4	6	4	6	6	6	6
938	12	0	0	0	0	12	0	12	12	12	12
1011	0	59	59	59	59	59	59	0	0	0	0
1013	3	5	3	3	3	5	3	3	3	3	3
1037	1	7	1	1	1	1	7	1	1	1	1
1054	1	8	1	1	1	1	8	1	1	1	1
1077	0	7	0	0	0	0	6	0	0	0	0
1104	1	1	1	4	4	1	1	1	1	1	1
1216	3	0	0	0	0	0	0	0	3	3	3
1252	5	5	1	1	1	1	5	5	5	5	5
1411	3	7	12	13	13	3	3	3	3	3	3

Table S3 - continued

CDS position in RLB1-8 (Reference)		Strains									
	RLB1-8	S1D4-23	RLB1-9	S1A1-3	S1A1-8	S1A1-7	RLB3-6	RLB3-17	RLB3-5	S1D4-14	S1D4-20
1425	103	95	172	175	175	95	95	18	103	103	103
1433	58	58	58	58	58	20	20	58	58	58	58
1523	20	20	20	20	20	20	20	8	20	20	20
1530	10	2	10	10	10	3	1	10	10	10	10
1532	3	3	0	0	0	3	3	3	3	3	3
1562	2	2	2	2	2	2	5	2	2	2	2
1597	7	7	7	7	7	7	7	0	7	7	7
1619	1	3	1	1	1	3	3	5	1	1	1
1630	4	6	4	4	4	4	4	4	4	4	4
1638	28	6	28	28	28	2	3	87	28	28	28
1642	2	2	5	5	5	2	2	2	2	2	2
1658	3	3	4	4	3	3	3	3	3	3	3
1672	0	0	6	5	5	0	0	0	0	0	0
1677	17	4	17	17	17	5	17	17	17	17	17
1687	1	1	4	4	4	1	1	1	1	1	1
1700	4	20	4	4	4	4	4	4	4	4	4
1713	4	4	4	4	4	11	12	11	4	4	4
1727	105	0	105	105	105	0	0	105	105	105	105
1737	12	12	12	12	12	12	12	12	12	12	12
1741	5	5	4	5	5	5	5	5	5	5	5
1745	5	1	5	5	5	1	2	5	5	5	5
1758	5	0	5	5	5	1	0	5	5	5	5
1829	2	2	2	2	2	2	2	11	2	2	2
1845	4	0	4	4	4	0	4	4	4	4	4
1876	3	3	2	2	2	27	38	5	3	3	3
1878	13	10	13	13	13	13	13	13	13	13	13
1903	0	4	0	0	0	0	4	0	0	0	0
2017	2	12	2	2	2	2	37	2	2	2	2

Table S3 - continued

CDS position in RLB1-8 (Reference)	Strains										
	RLB1-8	S1D4-23	RLB1-9	S1A1-3	S1A1-8	S1A1-7	RLB3-6	RLB3-17	RLB3-5	S1D4-14	S1D4-20
2067	4	3	11	11	11	11	7	4	4	1	1
2080	3	8	3	3	3	3	8	3	3	3	3
2153	12	14	12	12	12	12	14	12	12	12	12
2296	0	7	0	0	0	0	7	0	0	0	0
2359	2	2	2	2	2	4	2	2	2	2	2
2509	2	2	2	2	2	2	2	3	2	2	2
2614	0	3	0	0	0	0	3	0	0	0	0
2940	0	0	0	0	0	0	0	3	0	0	0
2977	41*	0	0	0	0	0	0	0	41*	41*	41*
3222	0	4	0	0	0	0	0	0	0	0	0
3485	0	0	20	20	20	20	0	0	0	0	0
3778	1	6	1	1	1	1	6	1	1	1	1
3785	0	4	0	0	0	0	4	0	0	0	0
3881	0	0	0	0	0	0	24*	0	0	0	0
3954	2	15*	2	2	2	17*	2	2	2	2	2
3956	0	0	0	0	0	0	0	0	0	0	84
4202	0	0	7	10	10	0	0	0	0	0	0
4252	3	3	3	3	3	5	3	3	3	3	3
4254	1	21*	1	1	1	1	21*	1	1	1	1
4351	0	7	0	0	0	0	7	0	0	0	0
4356	2	18*	2	2	2	2	4	2	2	2	2
4371	4	2	4	4	4	4	2	4	4	4	4
4380	8	6	8	8	8	8	6	8	8	8	8
4382	6	6	1	1	1	1	6	6	6	6	6
4384	4	4	4	4	4	4	4	0	4	4	4
4566	19	6	4	4	4	13	4	19	19	19	19

Table S3 - continued

CDS position in RLB1-8 (Reference)		Strains									
	RLB1-8	S1D4-23	RLB1-9	S1A1-3	S1A1-8	S1A1-7	RLB3-6	RLB3-17	RLB3-5	S1D4-14	S1D4-20
4916	3	22*	3	3	3	3	3	3	3	3	3
4927	0	4	0	0	0	0	4	20*	0	0	0
4971	1	6	1	1	1	1	6	1	1	1	1
4985	23	6	6	6	6	5	6	4	23	23	23
4992	11							2	11	11	11
5074	3	0	0	0	0	0	0	0	3	3	3
5250	0	0	0	0	0	0	18*	0	0	0	0
5270	0	11	0	0	0	11	0	0	0	0	0
5439	0	0	0	0	0	0	0	15*	0	0	0
5464	0	0	0	0	0	0	13*	0	0	0	31*
5476	3	16*	3	14*	16*	3	14*	22*	3	3	3
5797	5	4	5	5	5	5	4	5	5	5	5
5809	1	4	1	1	1	1	1	1	1	1	1
5946	0	0	0	0	0	27*	0	0	0	0	0
5991	85#	0	16*	16*	16*	0	0	0	85#	0	0
6080	7	0	0	2	2	0	0	0	7	7	7
6331	0	0	4	4	4	4	0	4	0	0	0
6375	0	0	18*	18*	18*	0	3	18*	0	18*	18*
6414	3	7	7	7	7	10*	7	7	3	3	3
6745	0	0	0	0	0	6	0	0	0	0	0
6807	4	2	2	2	2	4	2	2	4	4	4
7111	8	13*	8	8	8	14	13*	46*	8	8	8
7117	18*	18*	26*	29*	29 *	18*	18*	18*	18*	17*	17*
7127	8	8	8	8	8	9	8	9	8	8	8
7135	0	0	0	0	0	0	0	0	0	0	27

Table S3 - continued

CDS position in RLB1-8 (Reference)		Strains									
	RLB1-8	S1D4-23	RLB1-9	S1A1-3	S1A1-8	S1A1-7	RLB3-6	RLB3-17	RLB3-5	S1D4-14	S1D4-20
7232	3	3	3	3	3	3	3	3	3	3	5
7408	0	0	0	0	0	3	0	0	0	0	0
7441	1	3	1	1	1	1	3	1	1	1	1
7473	0	0	0	0	0	0	0	36*	0	0	0
7483	0	0	19*	19*	19*	0	15*	0	0	0	0
7663	3	1	1	1	1	1	1	1	3	3	3
7811	0	0	0	0	0	3	0	0	0	0	0
7945	1	1	1	1	1	1	4	1	1	1	1
7949	2	4	2	2	2	2	4	2	2	2	2
7977	24	11	2	2	2	12	11	5	24	24	24
8028	4	0	0	0	0	0	0	0	4	4	4
8059	0	0	0	0	0	17	0	0	0	0	0
8063	1	1	1	1	1	4	1	1	1	1	1
8114	1	1	1	1	1	5	1	1	1	1	1
8117	0	95	0	0	0	0	95	0	0	0	0
8183	0	241	139	138	138	53	241	144	0	0	0
8302	0	0	0	0	0	17	0	17	0	0	0
8390	0	0	4	4	4	0	0	4	0	0	0
8445	0	0	3	3	3	0	0	3	0	0	0
8452	3	3	5	5	5	3	3	5	3	3	3
8595	52	0	0	0	0	0	0	52	52	52	52
8687	0	0	27	27	27	0	0	31	0	0	0
8704	0	0	0	0	0	25*	0		0	0	0
9051	1	1	3	1	1	1	1	1	1	1	1
9063	1	3	1	1	1	1	3	1	1	1	1
9082	0	0	0	0	0	3	0	3	0	0	0
9139	4	4	4	4	4	4	4	5	4	4	4

Table S3 - continued

CDS position in RLB1-8 (Reference)		Strains									
	RLB1-8	S1D4-23	RLB1-9	S1A1-3	S1A1-8	S1A1-7	RLB3-6	RLB3-17	RLB3-5	S1D4-14	S1D4-20
9163	4	4	4	4	4	4	5	4	4	4	4
9362	2	6	2	2	2		6	2	2	2	2
9374	7	8	7	7	7	8	12	7	7	7	7
9400	6	0	6	6	6	0	0	6	6	6	6
9403	3	3	0	0	0	3	3	0	3	3	3
9409	1	1	1	1	1	1	3	1	1	1	1
9428	4	4	3	3	3	3	4	4	4	4	4
9435	7	7	7	7	7	7	10	7	7	7	7
9444	0	3	0	0	0	0	0	0	0	0	0
9610	4	1	4	4	4	4	1	4	4	4	4
9735	1		1	1	1	1	1	1	87	1	1
9831	1	34	1	1	1	1	32	1		1	1
9851	0	0	3	3	3	0	0	0	0	0	0
9962	2	5	7	7	7	5	5	7	2	2	2
9986	9	6	9	9	9	9	6	9	9	9	9
10169	2	4	2	2	2	7	4	2	2	2	2
10189	4	8	4	4	4	4	4	4	4	4	4
10192	1	1	1	1	1	1	6	1	1	1	1
10197	4	4	5	5	5	4	4	5	4	4	4
10211	3	0	0	0	0	0	0	0	3	3	3
10239	1	1	4	4	4	1	1	4	1	1	1
10254	10	10	2	2	2	10	10	2	10	10	10
10261	3	3	3	3	3	4	3	3	3	3	3
10283	14	2	14	14	14	2	2	14	14	14	14
10299	8	1	8	8	8	1	1	8	8	8	8
10325	1	1	1	1	1	1	1	5	1	1	1
10328	2	9	2	2	2	9	9	2	2	2	2
10337	5	5	5	5	5	10		5	5	5	5

Table S3 - continued

CDS position in RLB1-8 (Reference)		Strains									
	RLB1-8	S1D4-23	RLB1-9	S1A1-3	S1A1-8	S1A1-7	RLB3-6	RLB3-17	RLB3-5	S1D4-14	S1D4-20
10347	0	0	0	0	0	0	0	3	0	0	0
10351	3	3	3	3	3	12	3	3	3	3	3
10376	7	22	7	7	7	7	22	7	7	7	7
10400	2	2	2	2	2	5	2	2	2	2	2
10430	5	5	5	5	5	5	10	5	5	5	5
10441	1	1	1	1	1	1	7	1	1	1	1
10448	29	7	29	29	29	29	11	29	29	29	29
10470	2	2	2	4	2	2	2	2	2	2	2
10472	0	0	0	0	0	0	0	3	0	0	0
10487	4	4	4	4	4	2	7	4	4	4	4
10497	4	4	2	5	5	4	4	2	4	4	4
